# Brain-binding auto-antibodies in subjects with and without post-COVID-syndrome after SARS-CoV-2 infection

**DOI:** 10.1016/j.bbih.2026.101357

**Published:** 2026-09-18

**Authors:** Majd Faraj, Karin Weissenborn, Bianca Teegen, Meike Dirks, Ann-Katrin Hennemann, Vanessa Wuttke, Winfried Stöcker, Nina Gödecke, Rainer Blasczyk, Anika Großhennig, Hannelore Ehrenreich, Gernot Beutel

**Affiliations:** a Department of Neurology, Hannover Medical School, Hannover, 30623, Germany; b Clinical Immunological Laboratory Prof. Dr. med. Winfried Stoecker, Gross Groenau near, Lübeck, Germany; c Institute of Transfusion Medicine and Transplant Engineering, Hannover Medical School, Hannover, 30623, Germany; d Institute for Biostatistics, Hannover Medical School, Hannover, 30623, Germany; e Georg-August University, Göttingen, Germany; f Academic Teaching Practice of Internal Medicine, Hannover Medical School, Hannover, Germany

**Keywords:** Post-COVID syndrome, Post-COVID symptoms, Brain binding autoantibodies, N-Methyl-D-Aspartat-Receptor-1 (NMDAR1)-autoantibodies, COVID symptoms

## Abstract

Various pathomechanisms are discussed as possible causes of Post-COVID syndrome (PCS). Immune system dysfunction with increase of autoimmune responses is one of them. Whether the observed increase of autoantibody prevalence after COVID-19 is of any significance for the well-being of affected subjects in the long-term is controversial.

We assessed the seroprevalence of 44 autoantibodies against brain epitopes in 703 subjects a few months after a proven SARS-CoV-2 infection, and compared the results between 455 patients with Post-COVID syndrome and 248 subjects who had completely recovered. In addition, we looked for relationships between N-Methyl-D-Aspartate-Receptor-1 (NMDAR1)-autoantibody presence and symptoms during COVID-19 as well as Post-COVID symptoms in median 15.4 months after the infection, and we analyzed the occurrence of NMDAR1-autoantibodies regarding the severity of the PCS.

We observed a slight increase in serum autoantibody prevalence in our cohort compared to published data for healthy controls from the pre-pandemic time. Interestingly, NMDAR1 autoantibodies were by far the most frequent, with an occurrence twice as much as reported in the pre-pandemic literature. The increase in occurrence was predominantly based on an increase of NMDAR1-IgM antibodies.

We did not find differences in prevalence and features of auto-antibodies between patients with PCS and recovered individuals. Nor did we observe a relationship between the symptoms during COVID-19 or with PCS and the presence of serum autoantibodies.

Our data further support the conjecture of a dysregulated immune system after COVID-19, and endorse former observations that there is no direct link between autoantibody prevalence and clinical symptoms of Post-COVID syndrome.

## Highlights

• Seroprevalence of brain binding autoantibodies is increased in the long-term after COVID-19
• Post COVID Syndrome does not correlate with the prevalence of NMDAR1 autoantibodies
• Prevalence of NMDAR1 autoantibodies and COVID-19 features in the acute phase as well as in the long-term are unrelated
• Post COVID functional impairment is not related to presence of NMDR1 autoantibodies

## 1 Introduction

Despite intense efforts, the mechanisms behind the development of the Post-COVID-Syndrome (PCS) could not be clarified so far. Ongoing dysregulation of the innate immune system in the aftermath of the SARS-CoV-2 infection is considered one of the major possible causes of PCS (Altmann et al., 2023). Whether autoimmune reactions play a role is controversial (Sotzny et al., 2022; Bodansky et al., 2023; Ceccarini et al., 2023; Dobrowolska et al., 2023; Franke et al., 2023; Notarte et al., 2024; Almulla et al., 2025; Hofmann et al., 2025; Schmitz et al., 2025; de Sá et al., 2026). Some papers describe a significant relationship between the presence of distinct auto-antibodies and specific PCS symptoms while others could not find such a relationship.

Considering the neurological symptoms of PCS – which are the most frequent within the complex features of PCS - a possible role of brain-binding autoantibodies (AAB) is of utmost interest.

It should be kept in mind, however, that the role of autoantibodies (AAB) against brain epitopes in general is still not sufficiently understood. Are they an insignificant by-product of a working immune system with increasing prevalence with age? Do they cause neuropsychiatric diseases or do they just modify the course of neuropsychiatric diseases if present? The most frequent autoantibody against a brain epitope is the N-Methyl-D-Aspartate-Receptor-1 (NMDAR1)-autoantibody (Dahm et al., 2014; Zerche et al., 2015; Daguano Gastaldi et al., 2023). Dahm et al. (2014) described an increasing prevalence of NMDAR1-AAB with age, with about 10% seropositivity in younger adults compared to about 20% in the elderly. Blood-brain-barrier (BBB) function is considered to play a major role in the effects of brain-binding AAB. In case of a BBB alteration of any cause, brain epitopes are presented to the innate immune system resulting in the generation of the respective antibodies. These antibodies remain in the portfolio of B-lymphocytes without causing harm, unless BBB leakage occurs. In this case, antibody production will be persistently stimulated and the brain-binding AAB may cross the BBB and bind to their respective antigens, modulating their physiological function. Daguano Gastaldi et al. (2023) observed for example significantly lower serum NMDAR1-AAB presence in apolipoprotein E4 (APOE4) carriers - who are known to exhibit BBB dysfunction – compared to controls, and significantly higher prevalence in subjects with a history of repeated and/or severe neurotrauma but no indication for ongoing BBB damage.

BBB damage has been suspected as being part of the cerebral pathology in COVID-19 and PCS (Wallensten et al., 2024; Greene et al., 2024). Greene et al. (2024) presented data upon the interrelationship between BBB function and the presence of brain fog in PCS patients studied at a median of about 9 months after their acute disease. Using dynamic contrast-enhanced magnetic resonance imaging (DCE-MRI) they observed significantly increased whole-brain leakage in PCS patients with brain fog compared to those without and to recovered subjects. Considering the hypothesized interrelationship between BBB function and prevalence and functional impact of brain-binding AAB we assessed the prevalence of brain-binding AAB in serum of subjects with a history of recent SARS-CoV-2-infection with and without PCS to examine a possible correlation between presence of these antibodies and the chronification of neuropsychiatric symptoms after SARS-CoV-2-infection.

### 1.1 Subjects and methods

A total of 1599 subjects who had been infected with SARS-CoV-2 between February 2020 and May 2021 and had volunteered for plasma donation for the harvest of anti-SARS-CoV-2 antibodies were asked via e-mail to complete an online survey regarding their COVID-19 symptoms in the acute phase of the disease as well as their current wellbeing. In addition, they were asked to give their consent for use of any remaining blood samples for the assessment of the presence of brain-binding AAB. The survey took place between July 15 and August 15, 2021, with a follow-up in March 2024. This paper refers only to the data achieved by the first survey, which was completed by 992 of the 1599 invited subjects. At that time, none of the subjects had been infected for a second time. Blood samples for AAB assessment were available from 465 of the 992 subjects. In addition, blood samples from 148 patients with PCS who attended the Post-COVID outpatient clinic of Hannover Medical School between June 2021 and May 2023 and had been infected between March 2020 and December 2021 were analyzed. We also analyzed blood samples from 90 volunteers without persistent symptoms, who had experienced their first SARS-CoV-2 infection between March 2020 and August 2022. These samples were collected between March 2022 and December 2023. One-hundred-nineteen of the 148 patients from the outpatient clinic and 52 of the 90 recovered volunteers also completed the online survey.

All samples were collected after the acute phase of SARS-CoV-2 infection. For plasma donors, samples were collected approximately 6–12 months after infection. Among PCS outpatients, the median interval between SARS-CoV-2 infection and blood sampling was 10.5 months (IQR 7.3–16.2 months; range 3.8–33.0 months), compared with 11.4 months (IQR 7.6–25.1 months; range 2.4–45.1 months) among recovered volunteers.

The timing of SARS-CoV-2 infection also differed between the recruitment cohorts. Considering the date of infection and the predominant SARS-CoV-2 variants circulating in Germany, plasma donor participants were mainly infected during the wild-type and Alpha periods, whereas recovered volunteers were predominantly infected during the Omicron period. The distribution of presumed SARS-CoV-2 variant periods by recruitment cohort is shown in Supplementary Table S1.

The participant recruitment process is shown in Fig. 1A. Fig. 1B provides a schematic overview of the different recruitment sources and how the final study population was assembled.

**Fig. 1:**
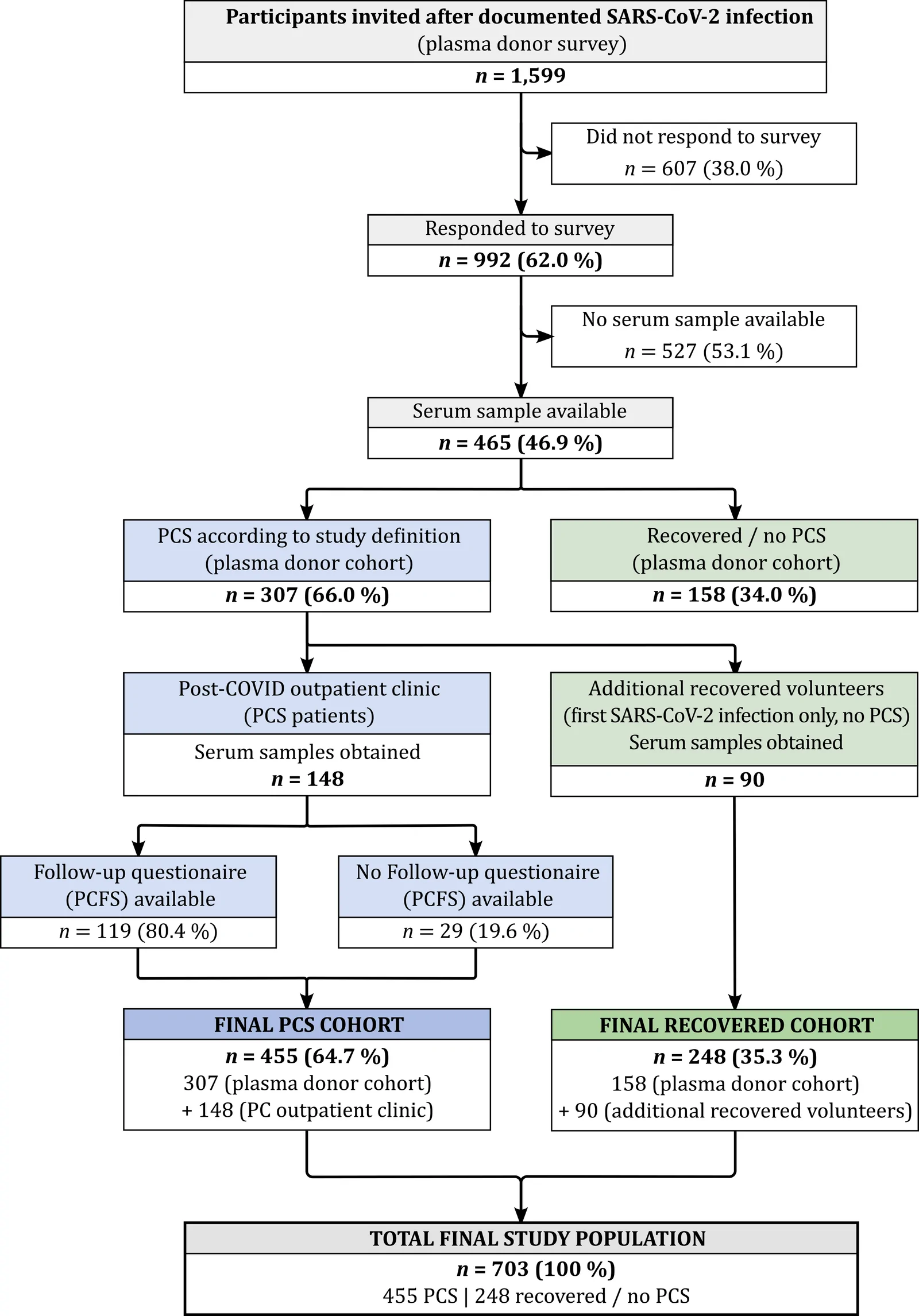

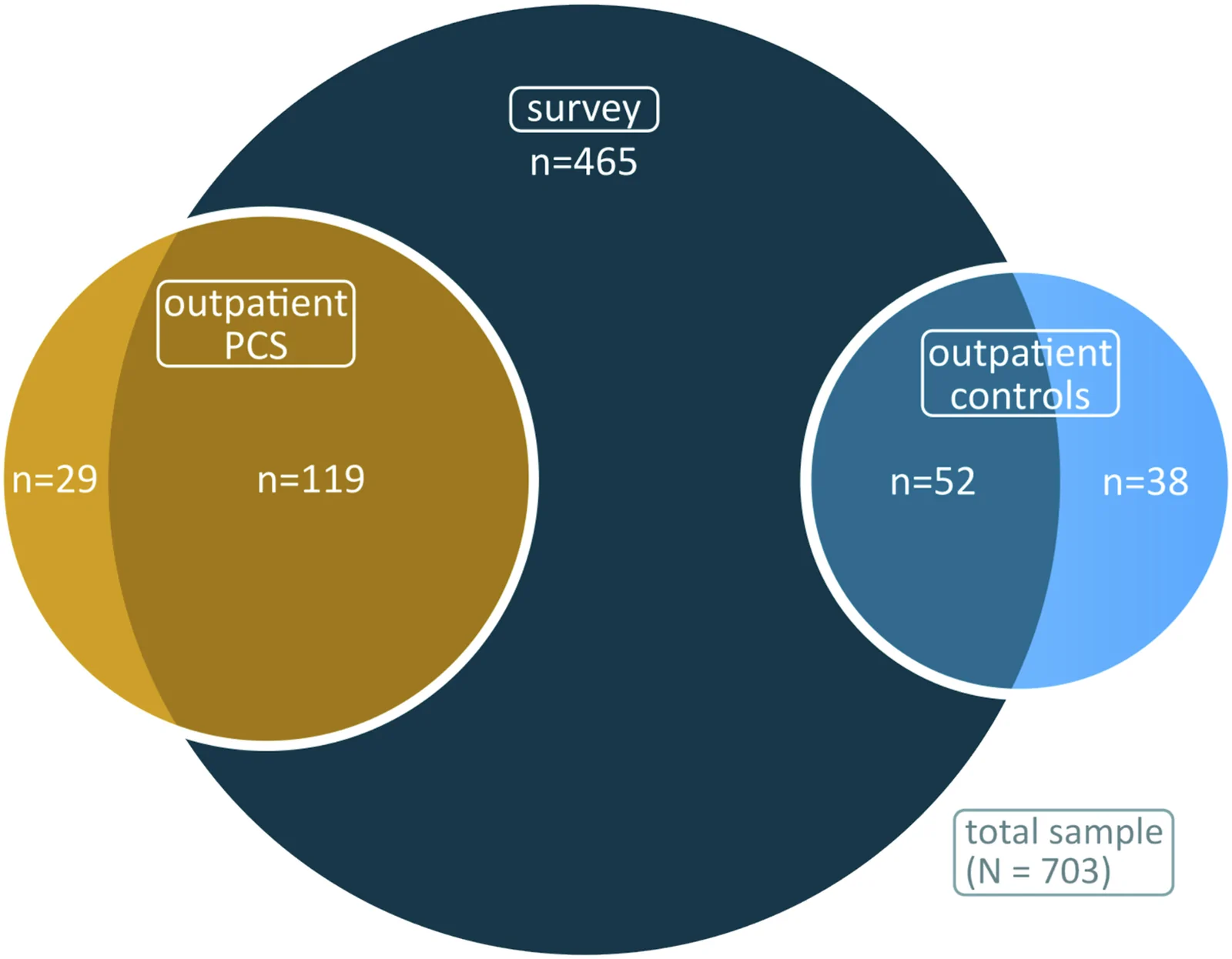
Study recruitment and assembly of the final study population. (A) Flow chart showing the recruitment of participants and the selection of the final study population. (B) Schematic overview of the overlap between the three recruitment sources and the composition of the final study population used for the different analyses.

The survey collected data on demographics (sex, age, ethnicity, height and weight), comorbidities, blood group, COVID-19 severity according to the World Health Organization (WHO) clinical progression scale, therapy, vaccination status, and a wide range of acute and persistent symptoms (neurological, psychiatric, cardiovascular, pulmonary, abdominal, musculoskeletal, endocrinologic, and others presented in Tables 2.1 and 2.2). Furthermore, current restrictions in daily life were assessed using the Post COVID-19 Functional Status scale (Klok et al., 2020) and additional information on reasons for SARS-CoV-2 testing, probable source of infection, and timing of infection and vaccination was requested. Information on the number of previous SARS-CoV-2 infections was collected as part of the survey and, for patients attending the outpatient clinic, during the structured medical interview. The survey questionnaire is shown in the supplement.

The diagnosis Post-COVID Syndrome was made retrospectively according to the WHO criteria, based on the subjects’ statements in the structured history taking via the survey or a personal interview. These criteria are considered fulfilled if a subject who suffered SARS-CoV-2 infection has ongoing symptoms for more than 3 months or develops new symptoms within this interval that cannot be explained by any other disease (https://www.who.int/europe/news-room/fact-sheets/item/post-covid-19-condition). The severity of the symptoms was classified according to the Post COVID-19 Functional Status Scale Score (PCFS) (Klok et al., 2020). which grades daily life limitations from 0 (no functional limitation) to 4 (severe functional limitation). For the present analyses, participants were grouped into those with no or negligible functional impairment (PCFS grades 0–1) and those with at least mild functional impairment (PCFS grades ≥2). This categorization identifies participants with clinically relevant limitations in daily functioning.

The study received a positive vote by the Ethics Committee of Hannover Medical School (9734_BO_K_2021) and was carried out in accordance with the World Medical Association Declaration of Helsinki. All participants signed an informed consent.

### 1.2 Serological analysis

Serological analyses were performed exactly as described previously by Dahm et al. (2014). Serum samples of all participants were tested for the presence of NMDAR1 AAB and a panel of further 43 anti-brain epitope-antibodies as follows: Biochip mosaics (Euroimmun, Lubeck, Germany) contained nonfixed nitrogen-frozen tissue cryosections (4 μm; rat hippocampus, monkey cerebellum) and recombinant cell substrates (formalin- or acetone-fixed recombinant HEK293 cells), each expressing a different neural antigen; namely NMDAR1, AMPAR1/R2, GABARB1/B2, LGI1, CASPR2, GAD65, mGluR1, mGluR5, Ma2, Recoverin, Zic-4, CARPVIII, Hu, Ri, CV2, Neurochondrin, Yo, ITPR1, Homer-3, Neurexin-3-alpha, ERC1, ARHGAP26, Tr/DNER, IgLON5, DPPX, AT1A3, GLRa1b, AQP4, GluRD2, MOG, GABA-a, Flotillin 1/2, KCNA1, MBP, DRD2, Contactin2, KCNA2, NF155, NF186, Contactin1, Sez6l2, AP3B2. Expression of the individual recombinant protein (autoantigen) was validated by immunological methods employing human or commercially available monospecific animal antibodies. Biochip mosaics were incubated each with 70 μl of phosphate-buffered saline (PBS)-diluted serum, starting at 1:10, for 30 min at room temperature, washed with PBS-Tween, and immersed in PBS-Tween for 5 min. Bound antibodies were stained with fluorescein isothiocyanate–labeled goat antihuman IgA, IgG, and IgM (Euroimmun) for 30 min at room temperature. Slides were washed again with a flush of PBS-Tween and then immersed in PBS-Tween for 5 min. Drops of PBS-buffered, 1,4-diazabicyclo[2.2.2]octane–containing glycerol (approximately 20 μl per field) were placed onto a cover glass, and the biochip slides were embedded in this mounting medium simultaneously and examined by fluorescence microscopy. Positive and negative controls were included with every test procedure. Samples were classified as positive or negative based on fluorescence intensity of the transfected cells in direct comparison with nontransfected cells and control samples.

The last dilution showing a measurable degree of fluorescence was considered as endpoint result, with 1:10 or 1:100 (depending on substrate) being the cutoff for positivity (Dahm et al., 2014).

### 1.3 Statistical analyses

This was an exploratory study. Categorical variables were compared between groups using χ^2^ tests or Fisher's exact tests to assess associations between clinical characteristics and autoantibody status. Specifically, for NMDAR1-AAB odds ratios (ORs) with 95% confidence intervals (CIs) were calculated from univariable logistic regression analyses using NMDAR1-negative subjects as the reference group.

In addition, a multivariable logistic regression analysis including age and sex as covariates was performed to assess the independent association between PCS status and NMDAR1 seropositivity.

NMDAR1-AAB seroprevalence was also compared between participants with slight (PCFS < 2) and moderate to severe functional impairment (PCFS ≥ 2).

Continuous variables are presented as mean ± standard deviation and were compared between groups using independent-samples t-tests. Two-sided p-values less than 0.05 were considered as significant. Given the exploratory nature of the symptom analyses, no formal correction for multiple testing was performed. The results were therefore interpreted with caution. Statistical analyses were performed using SPSS Statistics (version 30.0; IBM Corp., Armonk, NY, USA).

## 2 Results

Blood samples were available from 703 subjects. Of these, 455 fulfilled the criteria for a diagnosis of Post-COVID syndrome, while 248 had suffered COVID-19 without persistent sequelae (Table 1). None of the plasma donor participants and recovered volunteers had experienced more than one SARS-CoV-2 infection, whereas six individuals in the PCS outpatient cohort reported a reinfection. The Post-COVID patients were slightly older (44.8 ± 12.0 vs. 41.7 ± 12.9, independent-samples *t*-test, p = 0.002) and had a slightly higher proportion of female (63.5% vs 54%, p χ^2^ = 0.014) while groups did not significantly differ in the seroprevalence of NMDAR1-AAB. There was, however, a tendency for a higher prevalence of NMDAR1-IgM autoantibodies (AAB) in subjects without Post-COVID syndrome compared to those with persistent symptoms (13.7% vs 9.0% p χ^2^ = 0.054). The seroprevalence of all other antibodies measured was negligible and comparable between groups.

**Table 1:** Demographic characteristics and autoantibody seroprevalence in participants with and without Post-COVID syndrome.

Variables	Post-COVID N = 455	not Post-COVID N = 248	p-value
Age, mean (SD), y	44.8 (12.0)	41.7 (12.9)	**0.002**
Female sex, n (%)	289 (63.5%)	134 (54.0%)	**0.014**
Any AAB seropositive, n (%)	86 (18.9%)	53 (21.4%)	0.51
Non-NMDAR1 AAB, seropositive, n (%)	20 (4.4%)	11 (4.4%)	0.83
NMDAR1, seropositive, n (%)	70 (15.4%)	46 (18.5%)	0.28
NMDAR1-IgA, seropositive, n (%)	32 (7.0%)	17 (6.8%)	0.92
NMDAR1-IgG, seropositive, n (%)	9 (1.9%)	4 (1.6%)	0.73
NMDAR1-IgM, seropositive, n (%)	41 (9.0%)	34 (13.7%)	0.054
AMPAR1/R2, seropositive, n	0	0	
GABARB1/B2, seropositive, n	1	0	
LGI1, seropositive, n	0	0	
CASPR2, seropositive, n	2	3	
GAD65, seropositive, n	1	1	
mGluR1, seropositive, n	1	0	
mGluR5, seropositive, n	0	0	
Ma2, seropositive, n	0	0	
Recoverin, seropositive, n	1	1	
Zic-4, seropositive, n	0	0	
CARPVIII, seropositive, n	0	0	
Hu, seropositive, n	0	0	
Ri, seropositive, n	0	0	
CV2, seropositive, n	0	0	
Neurochondrin, seropositive, n	0	1	
Yo, seropositive, n	0	0	
ITPR1, seropositive, n	0	1	
Homer-3, seropositive, n	1	0	
Neurexin-3-alpha, seropositive, n	0	0	
ERC1, seropositive, n	0	0	
ARHGAP26, seropositive, n	0	1	
Tr/DNER, seropositive, n	0	0	
IgLON5, seropositive, n	1	0	
DPPX, seropositive, n	0	0	
AT1A3, seropositive, n	0	0	
GLRa1b, seropositive, n	3	0	
AQP4, seropositive, n	1	0	
GluRD2, seropositive, n	0	0	
MOG, seropositive, n	1	0	
GABA-a, seropositive, n	0	0	
Flotillin 1/2, seropositive, n	1	0	
KCNA1, seropositive, n	0	0	
MBP, seropositive, n	0	0	
DRD2, seropositive, n	0	0	
Contactin2, seropositive, n	0	0	
KCNA2, seropositive, n	3	2	
NF155, seropositive, n	1	0	
NF186, seropositive, n	1	0	
Contactin1, seropositive, n	0	0	
Sez6l2, seropositive, n	0	0	
AP3B2, seropositive, n	1	1	

Values are presented as number of seropositive subjects (n) or n (%), as indicated. Rare autoantibodies are shown as absolute counts, and no p-values were calculated due to low prevalence. Ig = immunoglobulin; yr = years; SD = standard deviation.

Because the groups differed with respect to age and sex, we additionally performed a multivariable logistic regression analysis including both variables as covariates. Adjustment for age and sex did not alter the main findings, as PCS status remained unrelated to NMDAR1 seropositivity (adjusted OR 0.75, 95% CI 0.49–1.15, p = 0.191). Increasing age was independently associated with NMDAR1 seropositivity (adjusted OR per year 1.03, 95% CI 1.01–1.05, p < 0.001), whereas sex showed no independent association (adjusted OR 0.73, 95% CI 0.49–1.10, p = 0.135) (Supplementary Table S2).

As participants were recruited from three different sources, we additionally compared autoantibody prevalences by recruitment source within both the PCS and recovered groups. Specifically, plasma donors with PCS were compared with the PCS outpatient cohort, whereas recovered plasma donors were compared with recovered volunteers. No significant differences in overall AAB prevalence, NMDAR1 seropositivity, or NMDAR1 immunoglobulin subclasses were observed between the two PCS cohorts. In contrast, recovered volunteers showed significantly higher prevalences of overall AAB, NMDAR1 seropositivity, and NMDAR1-IgM than recovered plasma donors (Supplementary Table S3).

A total of 636 subjects with brain AAB assessment completed the online survey, of whom 101 were positive for NMDAR1-AAB. Five-hundred-ninety-eight subjects had experienced only a mild or moderate course of COVID-19 without requiring hospitalization. A comparison of COVID-19-related symptoms between subjects with and without NMDAR1-AAB showed nominally significant differences only in the affection of smell and the presence of pneumonia. Disorders of smell had been more frequent in patients without NMDAR1-AAB in the aftermath, while subjects with NMDAR1-AAB had more frequently suffered from pneumonia during acute COVID-19 (Table 2.1).

**Table 2.1:** Characteristics and COVID-19 related symptoms at the time point of SARS-CoV-2 infection (2020 -2021) in subjects with and without NMDAR1 antibody seropositivity.

Variable	NMDAR1-negative (n = 535)	NMDAR1-positive (n = 101)	OR	95% CI
Neurological Symptoms				
Fatigue	422 (78.9%)	77 (76.2%)	0.86	0.52 – 1.42
Concentration deficit	226 (42.2%)	35 (34.7%)	0.73	0.46 – 1.13
Memory deficit	146 (27.3%)	21 (20.8%)	0.70	0.42 – 1.17
Dizziness	140 (26.2%)	19 (18.8%)	0.65	0.38 – 1.12
Light-headedness	80 (15.0%)	16 (15.8%)	1.07	0.60 – 1.92
Disorientation	20 (3.7%)	4 (4.0%)	1.06	0.36 – 3.17
Confusion	29 (5.4%)	3 (3.0%)	0.53	0.16 – 1.79
Hallucinations	4 (0.7%)	1 (1.0%)	-	-
Loss of consciousness	3 (0.6%)	1 (1.0%)	-	-
Headache	338 (63.2%)	54 (53.5%)	0.67	0.44 – 1.03
Neck stiffness	18 (3.4%)	1 (1.0%)	-	-
Sleep disturbance	132 (24.7%)	22 (21.8%)	0.85	0.51 – 1.42
Nightmares	34 (6.4%)	11 (10.9%)	1.80	0.88 – 3.69
Tremor	54 (10.1%)	9 (8.9%)	0.87	0.42 – 1.83
Seizure	4 (0.7%)	0 (0.0%)	-	-
Visual disturbance	56 (10.5%)	8 (7.9%)	0.74	0.34 – 1.59
Conjunctivitis	27 (5.0%)	4 (4.0%)	0.78	0.27 – 2.27
Olfactory dysfunction	323 (60.4%)	45 (44.6%)	**0.53**	**0.34 – 0.81**
Taste disturbance	328 (61.3%)	53 (52.5%)	0.70	0.45 – 1.07
Dysphagia (swallowing difficulty)	33 (6.2%)	8 (7.9%)	1.31	0.59 – 2.92
Speech disturbance	42 (7.9%)	8 (7.9%)	1.01	0.46 – 2.22
Movement disorder	35 (6.5%)	8 (7.9%)	1.23	0.55 – 2.73
Gait disturbance	29 (5.4%)	4 (4.0%)	0.72	0.25 – 2.09
Paralysis	4 (0.7%)	1 (1.0%)	-	-
Numbness/paresthesia	39 (7.3%)	8 (7.9%)	1.09	0.50 – 2.42
**Cardiovascular Symptoms**				
Palpitations	106 (19.8%)	17 (16.8%)	0.82	0.47 – 1.44
Irregular heartbeat	59 (11.0%)	8 (7.9%)	0.69	0.32 – 1.50
Chest tightness	110 (20.6%)	16 (15.8%)	0.73	0.41 – 1.29
Dyspnea at rest	92 (17.2%)	21 (20.8%)	1.26	0.74 – 2.15
Dyspnea on exertion	198 (37.0%)	28 (27.7%)	0.65	0.41 – 1.04
Thrombosis	4 (0.7%)	1 (1.0%)	-	-
Hypertension	40 (7.5%)	10 (9.9%)	1.36	0.66 – 2.82
Hypotension	21 (3.9%)	6 (5.9%)	1.55	0.61 – 3.93
**Respiratory Symptoms**				
Runny nose	154 (28.8%)	20 (19.8%)	0.61	0.36 – 1.03
Nasal congestion	140 (26.2%)	26 (25.7%)	0.98	0.60 – 1.59
Sore throat	254 (47.5%)	41 (40.6%)	0.76	0.49 – 1.16
Cough	303 (56.6%)	49 (48.5%)	0.72	0.47 – 1.10
Wheezing	42 (7.9%)	5 (5.0%)	0.61	0.24 – 1.59
Shortness of breath	138 (25.8%)	32 (31.7%)	1.33	0.84 – 2.12
Pneumonia	24 (4.5%)	13 (12.9%)	3.15	**1.54 – 6.41**
**Gastrointestinal and Genitourinary Symptoms**				
Nausea	85 (15.9%)	12 (11.9%)	0.71	0.37 – 1.36
Vomiting	25 (4.7%)	6 (5.9%)	1.29	0.51 – 3.23
Loss of appetite	156 (29.2%)	33 (32.7%)	1.18	0.75 – 1.86
Abdominal pain	57 (10.7%)	11 (10.9%)	1.02	0.52 – 2.03
Diarrhea	120 (22.4%)	15 (14.9%)	0.60	0.34 – 1.08
Constipation	5 (0.9%)	2 (2.0%)	-	-
Hair loss	59 (11%)	10 (10%)	-	-
Skin rash	52 (9.7%)	4 (4.0%)	0.38	0.14 – 1.08
**Musculoskeletal and Other Symptoms**				
Muscle weakness	104 (19.4%)	21 (20.8%)	1.09	0.64 – 1.84
Myalgia (muscle pain)	228 (42.6%)	36 (35.6%)	0.75	0.48 – 1.16
Arthralgia (joint pain)	190 (35.5%)	35 (34.7%)	0.96	0.62 – 1.50
Joint swelling	15 (2.8%)	1 (1.0%)	-	-
Lymphadenopathy	36 (6.7%)	5 (5.0%)	0.72	0.28 – 1.89
Leg edema	22 (4.1%)	5 (5.0%)	1.21	0.45 – 3.29
Tendency to fall	10 (1.9%)	2 (2.0%)	1.06	0.23 – 4.91
Back pain	130 (24.3%)	22 (21.8%)	0.87	0.52 – 1.45
**Psychiatric Symptoms**				
Anxiety disorder	76 (14.2%)	12 (11.9%)	0.81	0.43 – 1.56
Depressive symptoms	49 (9.2%)	8 (7.9%)	0.85	0.39 – 1.86

**(**NMDAR1-AAB-negative: N = 535; NMDAR1-AAB-positive: N = 101).

Values are presented as n (%). Odds ratios (ORs) with 95% confidence intervals (CIs) were derived from univariable analyses using NMDAR1-negative subjects as the reference group. ORs were not estimated for symptoms with very low event numbers.

Similar results for olfactory dysfunction were observed comparing the persistent symptoms of NMDAR1-AAB-positive and - AAB-negative subjects at the follow-up in 2021 - a median of 15.8 months (IQR, 9.2–16.4; range, 2.8–33.5 months) after their SARS-CoV-2 infection (Table 2.2). Of note, hypotension was more frequent now in subjects with NMDAR1-AAB.

**Table 2.2:** Characteristics and symptoms at a median follow-up of 15.8 months according to NMDAR1-AAB-Status (2021).

Variable	NMDAR1-negative n (%)	NMDAR1-positive n (%)	OR	95% CI
Neurological Symptoms				
Fatigue	236 (44.1%)	39 (38.6%)	0.80	0.52–1.23
Concentration deficit	168 (31.4%)	28 (27.7%)	0.84	0.52–1.34
Memory deficit	136 (25.4%)	19 (18.8%)	0.68	0.40–1.16
Dizziness	58 (10.8%)	10 (9.9%)	0.90	0.45–1.83
Light-headedness	19 (3.6%)	7 (6.9%)	2.02	0.83–4.94
Disorientation	13 (2.4%)	3 (3.0%)	1.23	0.34–4.39
Confusion	16 (3.0%)	3 (3.0%)	0.99	0.28–3.47
Hallucinations	2 (0.4%)	0 (0.0%)	–	–
Headache	95 (17.8%)	17 (16.8%)	0.94	0.53–1.65
Sleep disturbance	111 (20.7%)	17 (16.8%)	0.77	0.44–1.36
Nightmares	19 (3.6%)	6 (5.9%)	1.71	0.67–4.40
Tremor	14 (2.6%)	2 (2.0%)	0.75	0.17–3.36
Visual disturbance	38 (7.1%)	8 (7.9%)	1.13	0.51–2.49
Olfactory dysfunction	109 (20.4%)	12 (11.9%)	**0.53**	**0.28–0.99**
Taste disturbance	81 (15.1%)	13 (12.9%)	0.83	0.44–1.55
Dysphagia (swallowing difficulty)	8 (1.5%)	0 (0.0%)	–	–
Speech disturbance	34 (6.4%)	5 (5.0%)	0.77	0.29–2.01
Gait disturbance	16 (3.0%)	3 (3.0%)	1.00	0.28–3.47
Paralysis	6 (1.1%)	1 (1.0%)	0.88	0.10–7.82
Numbness/paresthesia	37 (6.9%)	9 (8.9%)	1.32	0.61–2.86
**Cardiovascular Symptoms**				
Palpitations	60 (11.2%)	13 (12.9%)	1.17	0.61–2.22
Irregular heartbeat	39 (7.3%)	7 (6.9%)	0.95	0.41–2.18
Chest tightness	46 (8.6%)	4 (4.0%)	0.44	0.15–1.25
Dyspnea at rest	22 (4.1%)	5 (5.0%)	1.21	0.45–3.29
Dyspnea on exertion	108 (20.2%)	18 (17.8%)	0.86	0.49–1.49
Thrombosis	2 (0.4%)	0 (0.0%)	–	–
Hypertension	48 (9.0%)	12 (11.9%)	1.37	0.69–2.68
Hypotension	11 (2.1%)	9 (8.9%)	**4.66**	**1.88–11.56**
**Respiratory Symptoms**				
Runny nose	23 (4.3%)	3 (3.0%)	0.69	0.20–2.32
Nasal congestion	25 (4.7%)	6 (5.9%)	1.29	0.52–3.21
Sore throat	23 (4.3%)	2 (2.0%)	0.45	0.10–1.94
Cough	41 (7.7%)	7 (6.9%)	0.89	0.39–2.06
Wheezing	13 (2.4%)	2 (2.0%)	0.81	0.18–3.65
Shortness of breath	61 (11.4%)	13 (12.9%)	1.15	0.60–2.18
**Gastrointestinal and Genitourinary Symptoms**				
Nausea	21 (3.9%)	6 (5.9%)	1.55	0.60–3.93
Vomiting	2 (0.4%)	1 (1.0%)	2.66	0.24–29.93
Loss of appetite	11 (2.1%)	2 (2.0%)	0.96	0.21–4.40
Abdominal pain	21 (3.9%)	8 (7.9%)	2.11	0.90–4.90
Diarrhea	26 (4.9%)	7 (6.9%)	1.46	0.61–3.46
Constipation	11 (2.1%)	2 (2.0%)	0.96	0.21–4.40
**Musculoskeletal and other Symptoms**				
Skin rash	28 (5.2%)	5 (5.0%)	0.94	0.35–2.50
Muscle weakness	50 (9.3%)	9 (8.9%)	0.95	0.45–1.99
Myalgia (muscle pain)	95 (17.8%)	14 (13.9%)	0.74	0.40–1.37
Arthralgia (joint pain)	82 (15.3%)	16 (15.8%)	1.04	0.58–1.86
Joint swelling	13 (2.4%)	1 (1.0%)	0.40	0.05–3.10
Lymphadenopathy	7 (1.3%)	3 (3.0%)	2.31	0.60–9.08
Leg edema	12 (2.2%)	4 (4.0%)	1.80	0.57–5.69
Tendency to fall	7 (1.3%)	1 (1.0%)	0.75	0.09–6.20
Back pain	57 (10.7%)	8 (7.9%)	0.72	0.33–1.56
**Psychiatric Symptoms**				
Anxiety disorder	46 (8.6%)	4 (4.0%)	0.44	0.15–1.25
Depressive symptoms	56 (10.5%)	8 (7.9%)	0.74	0.34–1.61

(NMDAR1-AAB-negative: N = 535; NMDAR1-AAB-positive: N = 101).

Values are presented as n (%). Odds ratios (ORs) with 95% confidence intervals (CIs) were derived from univariable analyses using NMDAR1-negative subjects as the reference group. ORs were not estimated for symptoms with very low event numbers.

In a further analysis we employed the Post-COVID Functional Status Score (PCFS) and compared the NMDAR1-AAB prevalence between Post-COVID patients with slight (PCFS < 2) and those with moderate to severe symptoms (PCFS ≥ 2). Patients with PCFS < 2 are not limited in their daily life, while patients with PCFS ≥ 2 suffer from moderate to severe limitations. Again, we found no significant group differences (Table 3). Table S4 in the supplement shows the distribution for all PCFS grades.

**Table 3:** Seroprevalence of NMDAR1-Antibodies by PCFS Category (<2 vs. ≥2).

	PCFS ≥ 2 (n = 167)	PCFS < 2 (n = 259)	P-Value
NMDAR1, seropositive, n (%)	23 (14%)	41 (16%)	0.562
NMDAR1-IgA, seropositive, n (%)	12 (7.2%)	17 (6.6%)	0.804
NMDAR1-IgG, seropositive, n (%)	1 (0.6%)	6 (2.3%)	0.16*
NMDAR1-IgM, seropositive, n (%)	16 (9.6%)	22 (8.5%)	0.701

Values are presented as n (%). Group comparisons were performed using χ^2^ tests or Fisher's exact tests. P-values marked with an asterisk (*) are based on two-sided Fischer's exact tests. PCFS: Post-COVID Functional Status score. 29 patients could not be considered in this analysis because the PCFS score was not available.

## 3 Discussion

The present study analyses the occurrence of 44 serum AAB against brain epitopes in 455 patients with Post-COVID-Syndrome compared to 248 subjects with a history of COVID-19 but no persistent symptoms. The most frequently detected antibody was the NMDAR1-AAB with 16.5% seropositive samples. All other antibodies were rarely detected, appearing in only 4.4% of the samples overall. We did not find significant differences regarding brain-binding AAB occurrence and spectrum between subjects with and without PCS, nor any compelling relationship between their presence and symptoms during COVID-19 or Post-COVID-disease. The findings regarding a possible relationship between AAB antibody occurrence and disturbance of smell (upper respiratory tract) on the one hand and pneumonia (lower respiratory tract) on the other hand during the acute phase of COVID-19 need to be further evaluated. This holds true also for the observed relationship between NMDAR1-AAB presence and hypotonia in the long-term. All these observations should be considered with caution as they relate to small numbers and are marginally significant. Nevertheless the observation of an increased prevalence of hypotension in NMDAR1-AAB-positive individuals is interesting considering recent discussions about a role of NMDAR in blood pressure regulation (Soda et al., 2023).

Dysregulation of the innate immune system resulting in persistent inflammation is discussed as one of the possible causes of the development and persistence of the Post-COVID syndrome (Dobrowolska et al., 2023; Moen et al., 2025). Whether autoimmune reactions play a role is controversial. A huge number of AAB that can be observed in healthy controls in less than 10% of the subjects have been found in more than half of the patients (some in > 90%) weeks to months after COVID-19, sometimes positively correlated with the course severity of the acute disease (Ceccarini et al., 2023; Dobrowolska et al., 2023). Whether these AAB are related to the risk for and development of Post-COVID-Syndrome remained unclear due to small numbers, missing controls, missing phenotyping of the patients and missing follow-up. There are a few AAB, however, that are repeatedly discussed playing a role in the development of PCS. Adrenergic and muscarinic G-protein-coupled-receptor AAB which have been frequently detected in patients with PCS have been considered possible drivers especially of cardiovascular PCS symptoms but also of neuropsychiatric symptoms (Wallukat et al., 2021; Seibert et al., 2023; Ceccarini et al., 2023; Schmitz et al., 2025; Hofmann et al., 2025). Also, Rodriguez-Perez and colleagues (2025) found increased levels of AAB targeting the pro-inflammatory Angiotensin II-Type 1 receptors in patients with neurological PCS compared to subjects with COVID-19 history but no ongoing symptoms and uninfected controls. AT1-receptor AAB correlated with Neurofilament light chain levels and cognitive impairment (Rodriguez-Perez et al., 2025). These findings, however, are in contrast to those presented by Sotzny et al. before (2022). Most recently, de Sá et al. (2026) looked for a causal link between IgG autoantibodies and neurological symptoms in patients with Long COVID (LC) as they termed it by analyses of blood samples from LC patients and recovered patients on average 416 days after the acute symptoms compared to healthy controls. They observed increased levels of a number of G protein-coupled receptors (GPCRs) related autoantibodies but no close relationship between the presence of distinct AAB and neurological symptoms. Of note they did not find a significant difference between recovered and LC patients regarding the prevalence of IgG NMDAR1-AAB, which is in accordance with our data, but the level of IgG NMDAR1-AAB was significantly higher in LC patients than in healthy controls.

Though there are some interesting leads, finally no clear relationship between feature and extent of clinical symptoms and distinct AAB could be unequivocally shown, so far (Klein et al., 2023; Bodansky et al., 2023; de Sá et al., 2026). As highlighted by Notarte et al. (2024), the available evidence is highly heterogeneous, with differences in study populations, methodologies, the investigated autoantibodies, and follow-up duration likely contributing to the discrepant findings across studies. In addition, differences in the interval between SARS-CoV-2 infection and blood sampling should be taken into account when comparing our findings with previous studies, as sampling time may influence the observed autoantibody levels.

There are only few studies dealing with AAB against brain cell epitopes in patients with PCS besides the study of de Sa et al. mentioned above. Vogelgesang and colleagues (2025) observed a correlation between levels of IgG targeting the 3 brain autoantigens DAB1 (disabled homolog 1), AIFM1 (apoptosis-inducing factor 1), and SURF1 (surfeit locus protein 1) and the Post COVID Functional Status (PCFS) as well as the frequency of self-reported respiratory symptoms in a cohort of PCS and no-PCS subjects with a history of SARS-CoV-2 infection. Almulla et al. (2025) report elevated levels of AAB against MBP (myelin basic protein), MOG (myelin oligodendrocyte glycoprotein), tubulin, CP2 (cerebellar protein 2), and synapsin in PCS patients. Moreover, they describe a positive correlation between IgM/IgA-synapsin, IgA/IgG-MBP, IgG-MOG, and CRP and AOPP (advanced oxidation protein products) levels and the extent of chronic fatigue, anxiety and depression in the PCS patients. Unfortunately, they did not include subjects with a history of SARS-CoV-2 infection but no ongoing symptoms into their study. The same shortcoming holds true for the study on brain-binding AAB in serum and cerebrospinal fluid of 50 subjects, who complained about cognitive dysfunction in median 250 days after SARS-CoV-2 infection, reported by Franke and colleagues (2023). Eighteen of the 50 subjects achieved abnormal results in the Montreal Cognitive Assessment (MoCA ≤ 25 points). Twelve patients had antibodies against myelin in serum, 2 Yo autoantibodies and 1 each GAD 65, Ma2/TA and NMDAR1 autoantibodies. Presence of antineuronal AAB in CSF was demonstrated using indirect immunofluorescence staining on mouse brain section in 16 of 50 patients. Patients with CSF autoantibodies achieved significantly lower MoCA scores than those without any antibodies in serum or CSF as well as - though less significant - those with AAB in serum but not CSF. Group comparisons were done using ANOVA without adjustment for age. This impedes the interpretation of the results as the prevalence of AAB in general increases with age, a finding that has been shown also for the sub-set of brain-binding AAB in large cohorts (Dahm et al., 2014, Daguano Gastaldi et al., 2023). Moreover, considering the rarity of auto-antibodies as a whole it is difficult to draw any conclusions based on the assessment of small patient cohorts. Of note, Daguano Gastaldi et al. (2023) found brain-binding AAB in about 16% of more than 7000 subjects, about 2700 of them being healthy controls. These results align with our findings of 18.9% and 21% in subjects with and without PCS. Daguano Gastaldi et al. (2023) did not find a significant difference in autoantibody presence between patients with different neuropsychiatric diseases and healthy controls. What they found was a significant increase of seropositivity with age, especially for NMDAR1-AAB. Gender was interrelated with the presence of non-NMDAR1 antibodies, with significantly higher rates in male subjects.

NMDAR1-AAB were detected in their study in 6.69% of 2.735 healthy controls and in 8.28% of 4.265 patients with various neuropsychiatric diseases. We found NMDAR1-AAB in 15.4% of our PCS cohort, and in 18.5% of the non-PCS cohort. Of interest, it is especially the prevalence of IgM antibodies against NMDAR1 that is higher in both groups of this study than that reported by Daguano Gastaldi et al., 2023) (Table 1). Both groups in our study – PCS and non-PCS - are of similar age as the complete sample analyzed by Daguano Gastaldi et al. (2023). Thus, age can be excluded as possible cause of the higher antibody prevalence. The finding could be an artifact due to the smaller numbers of subjects included in the present paper compared to the Daguano paper. The lower the frequency of an event the higher the number of subjects that must be studied to find out the real prevalence. However, the finding of increased AAB levels in PCS patients fits well with the respective literature (Needham et al., 2022; Altmann et al., 2023; Bodansky et al., 2023). Another possible cause that had to be considered is a - even unrecognized - change in the method used for the AAB assessment. This was clearly excluded by the colleagues in charge for the assessment. Nevertheless, additional NMDAR1-AAB analyses were performed on serum samples from 100 blood donors from 2014 and 140 blood donors from 2024 to validate the method. The antibody frequency in the 2014 cohort resembled that shown before by Dahm et al. (2014) or Daguano Gastaldi et al. (2023). In contrast, IgM NMDAR1-AAB were more frequent in the 2024 samples than in the 2014 samples (11.4% vs. 7%) and similar to the PCS and non-PCS groups in the present study, while there was no difference regarding IgA (7.8 vs.7%) and IgG (0,7 vs 1%) AAB. In contrast to IgM NMDAR1-AAB prevalence, IgA and IgG NMDAR1-AAB prevalence did not differ between the Daguano cohort and our cohort. The data point to an unspecific upregulation of autoantibody production in the aftermath of SARS-CoV-2 infection. Whether the increase in IgM NMDAR1 antibody prevalence in contrast to IgA and IgG antibody prevalence is of any clinical importance remains unclear. Considering the data presented by Needham and coworkers (2022), however, it seems worthwhile to follow this lead. Needham and colleagues observed in convalescent patients about 4 months after the SARS-CoV-2 infection increased numbers of IgG and IgM hits as well as a relationship between brain injury biomarker levels NfL, GFAP and total tau and IgM presence. So far, the cause for the increased AAB production even months after the SARS-CoV-2 infection is unclear. In regard to AAB against brain-antigens the persistent alteration of the blood-brain-barrier (Greene et al., 2024) might play a role. In this scenario the increased prevalence of brain-binding AAB could be considered as a response of the adaptive immune system to the persistent presentation of normally hidden brain epitopes. However, the increased NMDAR1-IgM presence months after COVID-19, especially in those subjects who did not develop PCS might also be due to a persistent activation of the innate immune system with production of natural IgM without class-switching to pathogenic IgG, thereby maintaining immune homeostasis and prevent chronic inflammation.

After transient BBB disruption, central neo-epitopes and apoptotic membrane fragments are presented and trigger innate immune responses (Hiéronimus and Huaux, 2023). In this context, a special role is attributed to B-1 lymphocytes, which mediate T-cell-independent responses by secreting protective IgM, IL-10, and GM-CSF. This allows to clear viral particles and danger-associated molecular patterns (DAMPs), thereby dampening severe inflammatory cytokine cascades (Aziz et al., 2020; Hiéronimus and Huaux, 2023). Furthermore, secreted IgM serves as an activator of the classical opsonophagocytic complement cascade and prevents early systemic inflammation (Aziz et al., 2020; Macela and Kubelkova, 2021; Hiéronimus and Huaux, 2023).

Our findings show a higher NMDAR1-IgM seroprevalence in recovered subjects compared to PCS patients, supporting the hypothesis that natural IgM protects against persistent clinical disease by acting as an immunomodulatory scavenger and restoring immunohomeostasis.

In fact, there is increasing evidence that so-called natural IgM (and also IgG) have a protective role in human infectious disorders, cancer, cardiovascular diseases and also neurodegenerative disorders (Reyneveld et al., 2020). In this context, the higher prevalence of NMDAR1-IgM in recovered individuals without persistent symptoms could be compatible with a physiological, homeostatic immune response rather than a pathogenic autoimmune process.

## 4 Limitations

The present study includes substantially more subjects than any other study focused on brain-binding AAB in the long-term after SARS-CoV-2 infection before. Nevertheless, higher numbers would have been of value to back up our findings and conclusions. Further limitations are the heterogeneous recruitment from three different sources, the reliance on self-reported symptoms and re-infection history, and the absence of a healthy non-COVID control group. Furthermore, the SARS-CoV-2 infections in our cohort occurred between February 2020 and August 2022. Thus, it must be considered that the different virus strains present throughout this time interval might have had different influence upon immune reactions and antibody profiles.

In addition, it must be emphasized that the present study is limited to the detection of a few AAB using a commercially available diagnostic assay. Our finding of a missing relationship between AAB occurrence and feature and extent of clinical symptoms, both during COVID-19 as well as thereafter, using this assay does not exclude a relationship between autoimmune reactions and the development of PCS, in general. There are thousands of brain antigens that have not been addressed in this study, most of them are even not yet characterized.

## 5 Conclusions

Autoantibodies targeting brain epitopes are more frequent in subjects who suffered SARS-CoV-2 infection compared to pre-pandemic controls. None of the autoantibodies frequently analyzed for clinical diagnostic purposes showed different prevalence between COVID-19 survivors with and without PCS. IgM NMDAR1-AAB were twice as frequent as expected based on former studies (Dahm et al., 2014; Daguano Gastaldi et al., 2023), but not significantly related to feature and extent of symptoms in PCS patients.

## CRediT authorship contribution statement

**Majd Faraj:** Writing – review & editing, Writing – original draft, Visualization, Validation, Formal analysis, Data curation. **Karin Weissenborn:** Writing – review & editing, Writing – original draft, Validation, Supervision, Resources, Project administration, Methodology, Funding acquisition, Formal analysis, Data curation, Conceptualization. **Bianca Teegen:** Writing – review & editing, Validation, Methodology, Investigation. **Meike Dirks:** Writing – review & editing, Validation, Project administration, Methodology, Investigation, Data curation. **Ann-Katrin Hennemann:** Writing – review & editing, Validation, Methodology, Investigation, Data curation. **Vanessa Wuttke:** Writing – review & editing, Investigation, Data curation. **Winfried Stöcker:** Writing – review & editing, Resources, Methodology. **Nina Gödecke:** Writing – review & editing. **Rainer Blasczyk:** Writing – review & editing, Resources. **Anika Großhennig:** Writing – review & editing, Validation. **Hannelore Ehrenreich:** Writing – review & editing, Writing – original draft, Funding acquisition, Conceptualization. **Gernot Beutel:** Writing – review & editing, Writing – original draft, Validation, Software, Resources, Funding acquisition, Formal analysis, Data curation, Conceptualization.

## Ethics declaration

Written informed consent to take part in the study and to publish the article has been obtained from all participants or their legal representatives. The privacy rights of participants have been observed.

This study includes human biological material and consent was obtained by donors, or their next of kin or legal representatives, for use in this study and for publication of the article. The samples used in this research were not sourced from executed prisoners or prisoners of conscience.

This study was performed in compliance with relevant laws, regulatory frameworks and guidelines where the research took place. This study was approved by the Ethics Committee at Hannover Medical School. (Approval No. 9734_BO_K_2021)

## Submission declaration

The work described has not been published previously. It is not under consideration for publication elsewhere. Its publication is approved by all authors and tacitly or explicitly by the responsible authorities where the work was carried out. If accepted, it will not be published elsewhere in the same form, in English or in any other language, including electronically without the written consent of the copyright-holder.

## Declaration of generative AI and AI-assisted technologies in the manuscript preparation process

During the preparation of this work the author(s) used no generative AI and AI-assisted technologies.

## Funding

This work was supported by the Karlheinz-Hartmann-Foundation, Hannover, Germany.

## Declaration of competing interest

The authors declare that they have no known competing financial interests or personal relationships that could have appeared to influence the work reported in this paper.

## Data Availability

The authors do not have permission to share data.
