# Supplementary material for: Brain-binding auto-antibodies in subjects with and without post-COVID-syndrome after SARS-CoV-2 infection
Source: Brain Behav Immun Health. 2026 Sep 18;57:101357. doi: 10.1016/j.bbih.2026.101357 (PMC13625633; doi:10.1016/j.bbih.2026.101357)
Supplement: Supplementary file 1 [file mmc2.pdf]

ONLINE BEFRAGUNG ZUR RETROSPEKTIV-PROSPEKTIVEN ERFASSUNG DER SYMPTOMKOMPLEXE UND KRANKHEITSVERLÄUFE ÜBERWIEGEND AMBULANT BEHANDELTER COVID-19-PATIENT*INNEN RAHMEN DES LEOSS-PROJEKTES UND VERKNÜPFUNG KLINISCHER PARAMETER MIT DEM AUTOANTIKÖRPER-PROFIL GEGEN ZNS-EPITOPE

Sehr geehrte Damen und Herren,

Sie waren freundlicherweise unserem Aufruf gefolgt, sich nach durchstandener COVID-19- Erkrankung auf Antikörper gegen das die Erkrankung hervorrufende SARS-CoV-2-Virus testen zu lassen, und gegebenenfalls Blut-Plasma für die Antikörpergewinnung zu spenden. Dafür möchten wir Ihnen ganz herzlich danken.

Wir wenden uns heute mit der Bitte um weitere Unterstützung an Sie: Um genaueres Wissen über den Verlauf der Erkrankung zu erlangen, ist es notwendig, Patienten mit unterschiedlichsten Schweregraden der Erkrankung zu befragen. Die derzeit vorliegenden Daten stützen sich vor allem auf Befunde von Patienten mit schwerwiegenden Verläufen, die einen Krankenhausaufenthalt notwendig machten. Befunde von Patient*innen mit ambulanten Verläufen sind bislang wenig untersucht.

In den vergangenen Monaten ist deutlich geworden, dass die COVID-19-Erkrankung häufig mit neurologischen Beschwerden einhergeht (wie beispielsweise Geruchs- und Geschmacksstörung). Wir müssen davon ausgehen, dass das Virus vermutlich über den Riechnerv Zugang zum Gehirn hat, und dort eine direkte Entzündungsreaktion und / oder auch eine chronische Immunantwort auslöst. Von anderen viralen Erkrankungen wissen wir, dass es zu einer Zunahme der Bildung sogenannter Auto-Antikörper gegen Bestandteile von Hirnzellen kommen kann, welche die Funktion dieser beeinträchtigen. Uns interessiert nun, ob es auch bei der COVID-19-Erkrankung zu einer vermehrten Bildung solcher Auto-Antikörper kommt. Dies würde möglicherweise das Auftreten neurologischer Symptome im Langzeitverlauf erklären, von denen jetzt zunehmend berichtet wird. Wir möchten daher die von Ihnen geleistete Blutspende nicht nur auf das Vorhandensein von Antikörpern gegen das SARS-CoV-2-Virus untersuchen, sondern zusätzlich auf das Vorhandensein von Auto-Antikörpern gegen Bestandteile von Hirnzellen und Marker für Schäden an Gehirnzellen.

Die Bearbeitung des Fragenkatalogs können Sie unabhängig davon durchführen, ob Sie einer Ausweitung der Untersuchungen Ihrer Blutproben zustimmen oder nicht. Sie können auch isoliert der Erweiterung der Blutuntersuchungen zustimmen, ohne den Fragenkatalog zu beantworten. Allerdings lässt sich nur aus der Kombination von Beschwerdeschilderung und Ergebnis der Blutuntersuchungen schließen, ob und inwieweit die Auto-Antikörperbildung Einfluss auf den Krankheitsverlauf hat.
Ihre Blutproben werden pseudonymisiert analysiert, d.h. Sie können nicht ohne Weiteres Ihrer Person zugeordnet werden. Im Institut für Transfusionsmedizin ist ein Code hinterlegt, der mit Ihrem Namen verbunden ist und diese Zuordnung ermöglicht.

Mit Ihrer Studienteilnahme unterstützen Sie die Forschung zu Folgeerkrankungen von COVID19. Die Teilnahme an der Studie ist freiwillig. Ein direkter Nutzen für Sie persönlich ergibt sich derzeit nicht. Sie können jederzeit und ohne Angabe von Gründen die Zustimmung zur Teilnahme an dieser Studie widerrufen, ohne dass sich dies nachteilig auf Ihre weitere medizinische Betreuung auswirkt. Auf Wunsch werden dann alle für die Studie gespeicherten Daten gelöscht und alle noch vorhandenen Blut- und Gewebeproben vernichtet. Forschungsergebnisse, die bis dahin mit diesen Daten erzielt wurden, sind von diesem Widerruf nicht betroffen.

Wir danken Ihnen für Ihre Studienteilnahme und Unterstützung im Kampf gegen COVID-19!

Mit freundlichen Grüßen,

Prof. Dr. Karin Weißenborn, Prof. Dr. Rainer Blasczyk, Dr. Gernot Beutel

EINWILLIGUNG ZUM FRAGEBOGEN

- ○ JA, ich möchte den Fragebogen beantworten.
- ○ Nein, ich möchte nicht an der Befragung teilnehmen.

EINWILLIGUNG ZUR ANALYSE DER BLUTPROBEN

- ○ JA, ich bin mit der Untersuchung meiner Blutprobe einverstanden.
- ○ Nein, ich wünsche keine weitere Untersuchung meiner Blutprobe.
- ○ Nicht zutreffend, da ich keine Blutprobe abgegeben habe.

DATENSCHUTZ

In dieser Studie werden personenbezogene Daten erhoben, gespeichert und ausgewertet. Die Verarbeitung Ihrer Daten unterliegt der ärztliche Schweigepflicht und den Bestimmungen der Datenschutzgrundverordnung. Ihre Angaben werden ausschließlich pseudonymisiert gespeichert, d.h. ohne Nennung Ihres Namens und zusätzlich verschlüsselt durch einen Zahlencode. Auch auf etwaigen Blutproben wird lediglich ein Zahlencode angegeben, so dass eine einfache Rückverfolgung der Proben nicht möglich ist. Erst In Kombination mit einer separat gespeicherten Decodierungsliste kann auf Ihre personenbezogenen Daten rückgeschlossen und die Angaben aus der Umfrage mit den Ergebnissen aus den Blutuntersuchungen zusammengeführt werden. Sowohl Ihre Persönlichen Daten als auch Ihre Blutproben werden an einem sicheren Ort verwahrt, so dass ein Missbrauch vermieden wird. Dritte können durch das Pseudonyms nicht auf Ihre Person rückschließen. Zur Wahrung guter wissenschaftlichen Praxis werden die Daten für Forschungsprojekte für 10 Jahre aufbewahrt und im Anschluss gelöscht.

Die Teilnahme an der Studie ist freiwillig und unterstützt die Forschung zu Folgeerkrankungen von COVID19. Ein direkter Nutzen für Sie persönlich ergibt sich derzeit nicht. Sie können jederzeit und ohne Angabe von Gründen die Zustimmung zur Teilnahme an dieser Studie widerrufen, ohne dass sich dies nachteilig auf Ihre weitere medizinische Betreuung auswirkt. Auf Wunsch können alle für die Studie gespeicherten Daten gelöscht und die vorhandenen Blutproben vernichtet werden, soweit nicht gesetzliche Aufbewahrungsfristen entgegenstehen. Sie können Sich bei Ausscheiden aus der Studie entscheiden, ob Sie mit der Auswertung des Materials bzw. der Studiendaten einverstanden sind.

Gemäß Datenschutzgrundverordnung (DSGVO) und dem Bundesdatenschutzgesetz (BDSG) weisen wir Sie ausdrücklich auf die folgenden Rechte hin:
- Sie können jederzeit Auskunft über die Sie betreffenden personenbezogenen Daten, die im Rahmen dieser Studie erhoben, verarbeitet oder an Dritte übermittelt werden, verlangen (Artikel 15 DSGVO, §§34 und 57 BDSG).
- Sie haben das Recht, Sie betreffende unrichtige personenbezogene Daten berichtigen zu lassen (Artikel 16 und 19 DSGVO, §58 BDSG).
- Sie haben das Recht auf Löschung personenbezogener Daten, z.B. wenn diese Daten für den Zweck, für den sie erhoben wurden, nicht mehr notwendig sind (Artikel 18 und 19 DSGVO, §§ 35 und 58 BDSG).
- Sie haben das Recht auf Einschränkung der Verarbeitung, d.h. die Daten dürfen nur gespeichert, nicht verarbeitet werden. Wenden Sie sich hierzu bitte an Ihren/ Ihre Studienarzt/-ärztin (Artikel 18 und 19 DSGVO, § 58 BDSG).
- Sie haben das Recht, die Sie betreffenden personenbezogenen Daten, die Sie dem Verantwortlichen für diese Studie bereitgestellt haben, zu erhalten. Sie können beantragen, dass diese Daten entweder Ihnen oder, soweit technisch möglich, einer anderen von Ihnen benannten Stelle übermittelt werden (Artikel 20 DSGVO).
- Die Verarbeitung Ihrer personenbezogenen Daten ist nur mit Ihrer Einwilligung rechtmäßig (Artikel 6 DSGVO, § 51 BDSG).
- Sie haben das Recht, Ihre Einwilligung zur Verarbeitung personenbezogener Daten jederzeit zu widerrufen. Es dürfen jedoch die bis zu diesem Zeitpunkt erhobenen Daten durch die in der Patienteninformation und Einwilligungserklärung zu der jeweiligen Studie genannten Stellen verarbeitet werden (Artikel 7, Absatz 3 DSGVO, § 51 Absatz 3 BDSG).

Um die Pseudonymisierung in dieser Studie aufrechterhalten zu können, bitten wir Sie bei Inanspruchnahme eines der oben genannten Rechte, sich an Ihren Studienarzt zu wenden.

Bei allgemeinen Fragen zum Datenschutz können Sie sich an die Datenschutzbeauftragte der MHH wenden (Carl-Neuberg-Str. 1, 30625 Hannover, Telefon: 0511-532-2555). Außerdem haben Sie das Recht, Beschwerde bei der zuständigen Datenschutzaufsichtsbehörde einzulegen, wenn Sie der Ansicht sind, dass die Verarbeitung der Sie betreffenden personenbezogenen Daten gegen die DSGVO verstößt (Die Landesbeauftragte für den Datenschutz Niedersachsen, Prinzenstraße 5, 30159 Hannover, 0511-120-4500).

EINWILLIGUNG ZUM DATENSCHUTZ

- ○ JA, ich stimme den Datenschutzbestimmungen zu.
- ○ Nein, ich stimme nicht zu.

FREISCHALTUNGS-CODE

FREISCHALTUNGS-CODE

Mit Ihrem Einladungsschreiben haben wir Ihnen einen Code zur Freischaltung des Fragebogens übermittelt. Bitte tragen Sie diesen Code in das folgende Feld ein:

Freischaltungs-Code ____________________

ANGABEN ZUR PERSON

Bitte machen Sie Angaben zu Ihrem Geschlecht:

- ○ Männlich
- ○ Weiblich
- ○ Divers

Bitte geben Sie Ihre Körpergröße an:

Körpergröße [ ] cm

Bitte geben Sie Ihre Körpergröße an:

Körpergewicht [ ] kg

Wie alt waren Sie zum Zeitpunkt der COVID-19 Diagnose?

Alter [ ] Jahre

Bitte machen Sie Angaben zur Ihrer Herkunft?

[alphabetische Sortierung]

- ○ Amerikanisch
- ○ Asiatisch
- ○ Arabisch
- ○ Afro Amerikanisch
- ○ Europäisch
- ○ Sonstige, bitte angeben: [ ]
- ○ Unbekannt

Arbeiten Sie im Gesundheitswesen?

- ○ Ja, mit direktem Patientenkontakt (z.B. Krankenpflege, Physiotherapeut)
- ○ Ja, im Servicebereich (z.B. Raumpflege, Küche, Instandsetzung)
- ○ Ja, in einem Laborbereich ohne Patientenkontakt
- ○ Sonstige, bitte angeben: ______
- ○ Nein

Welche Blutgruppe haben Sie?

- ○ A
- ○ B
- ○ 0
- ○ AB
- ○ Unbekannt

Wie ist Ihr Rauchverhalten?

- ☐ Ich habe nie geraucht
- ☐ Ich habe früher geraucht
- ☐ Ich bin aktiver Raucher seit ______ Jahren
- ☐ Ich rauche ______ Schachteln pro Tag
- ☐ Unbekannt

ANGABEN ZUR SARS-COV-2 INFEKTION

Wann wurde bei Ihnen SARS-CoV-2 nachgewiesen?

[ ] (Format: TT.MM.JJJJ)

In welchem Zusammenhang wurde Ihre Diagnose gestellt?

- ○ Testung bei Krankheitsverdacht (Symptome)
- ○ Reihentestung bei Ausbruchsgeschehen
- ○ Kontaktpersonennachverfolgung nach Exposition
- ○ Routinetest vor medizinischer Behandlung (Krankenhaus)
- ○ Angeordneter Test bei erhöhtem Risiko (Einreise aus Risikogebiet)
- ○ Freiwillige Testung
- ○ Sonstige, bitte angeben: [ ]
- ○ Unbekannt

Bitte nennen Sie den Aufenthaltsort (möglichen Infektionsort) vor Nachweis von SARS-CoV-2:

- ○ Zu Hause
- ○ Krankenhaus
- ○ Reha-Einrichtung
- ○ Pflegeheim
- ○ Obdachlosigkeit
- ○ Flüchtlingsunterkunft
- ○ Sonstige, bitte angeben: [ ]

Handelt es sich um eine Erst- oder Zweitinfektion?

- ○ Erstinfektion
- ○ Zweitinfektion
- ○ Unbekannt

SYMPTOME BEI SARS-COV-2 INFEKTION

Im Folgenden möchten wir Sie zu Ihren Symptomen zum Zeitpunkt der Diagnose der COVID-19 Erkrankung befragen. Für den Fall, dass Sie symptomlos erkrankt sind, wählen Sie bitte die entsprechenden Antwortoptionen.

Zur Übersichtlichkeit sind die Symptome nach Organsystemen sortiert. Schauen Sie sich die Liste bitte in Ruhe durch, Mehrfachantworten sind möglich.

Wann haben Sie das erste Mal Symptome bemerkt?

- ○ Datum: ______ (Format: TT.MM.JJJJ)
- ○ Ich bin symptomfrei erkrankt

Sind bei Ihnen Allgemeinsymptome aufgetreten?

- ☐ Fieber / Schüttelfrost
- ☐ Nachtschweiß
- ☐ Verminderte körperliche Belastbarkeit
- ☐ Sonstige, bitte angeben: ______
- ☐ Es sind keine Allgemeinsymptome aufgetreten

SYMPTOME BEI SARS-COV-2 INFEKTION

Sind bei Ihnen Symptome des Nervensystems aufgetreten?

- ☐ Müdigkeit
- ☐ Schnelle Ermüdbarkeit
- ☐ Konzentrationsschwäche
- ☐ Gedächtnisstörung
- ☐ Schwindel
- ☐ Benommenheit
- ☐ Orientierungsstörung
- ☐ Verwirrtheit
- ☐ Halluzinationen
- ☐ Bewußtlosigkeit
- ☐ Kopfschmerzen
- ☐ Genickstarre (Meningismus)
- ☐ Schlafstörung
- ☐ Alpträume
- ☐ Zittern
- ☐ Krampfanfall
- ☐ Sehstörungen
- ☐ Gerötetes Auge (Bindehautentzündung)
- ☐ Geruchsstörung
- ☐ Geschmacksstörung
- ☐ Schluckstörungen
- ☐ Sprechstörungen
- ☐ Bewegungsstörungen
- ☐ Gangstörungen
- ☐ Lähmungen
- ☐ Taubheitsgefühle
- ☐ Schlaganfall
- ☐ Sonstige, bitte angeben: ____________
- ☐ Keine Symptome

SYMPTOME BEI SARS-COV-2 INFEKTION

Sind bei Ihnen Symptome des Herz und Gefäßsystems aufgetreten?

- ☐ Herzklopfen
- ☐ Herzstolpern
- ☐ Engegefühl in der Brust (Angina pectoris)
- ☐ Herzinfarkt
- ☐ Atemnot in Ruhe
- ☐ Atemnot bei Belastung
- ☐ (Beinvenen-) Thrombose
- ☐ Blutungen
- ☐ Hoher Blutdruck
- ☐ Niedriger Blutdruck
- ☐ Sonstige, bitte angeben: ______
- ☐ Keine Symptome

SYMPTOME BEI SARS-COV-2 INFEKTION

Sind bei Ihnen Symptome der Atemwege aufgetreten?

- ☐ Laufende Nase (Rhinorrhoe)
- ☐ Verstopfe Nase
- ☐ Halsschmerzen
- ☐ Husten
- ☐ Keuchen
- ☐ Luftnot
- ☐ Lungenentzündung
- ☐ Sonstige, bitte angeben: ______
- ☐ Keine Symptome

SYMPTOME BEI SARS-COV-2 INFEKTION

Sind bei Ihnen Symptome von Magen, Darm oder Blase aufgetreten?

- ☐ Übelkeit
- ☐ Erbrechen
- ☐ Appetitlosigkeit
- ☐ Bauchschmerzen
- ☐ Durchfall
- ☐ Verstopfung
- ☐ Stuhl-Inkontinenz
- ☐ Harnverhalt
- ☐ Harn-Inkontinenz
- ☐ Sonstige, bitte angeben: ____________
- ☐ Keine Symptome

SYMPTOME BEI SARS-COV-2 INFEKTION

Sind bei Ihnen Symptome von Haut, Muskel und Skelettsystem aufgetreten?

- ☐ Haarverlust
- ☐ Hautausschlag
- ☐ Muskelschwäche
- ☐ Muskelschmerzen
- ☐ Gelenkschmerzen
- ☐ Gelenkschwellung
- ☐ Geschwollene Lymphknoten
- ☐ Geschwollene Beine
- ☐ Sturzneigung
- ☐ Rückenschmerzen
- ☐ Sonstige, bitte angeben: ____________
- ☐ Keine Symptome

SYMPTOME BEI SARS-COV-2 INFEKTION

Sind weitere Symptome aufgetreten?

- ☐ Angststörung
- ☐ Depression
- ☐ Psychose
- ☐ Blutzuckerveränderungen
- ☐ Sonstige, bitte angeben: ______
- ☐ Keine weiteren Symptome

Seit dem folgendem Datum habe ich keine Symptome mehr:

- ○ Datum: ______ (Format: TT.MM.JJJJ)
- ○ Es bestehen weiterhin Symptome
- ○ Ich war symptomfrei erkrankt

ANGABEN ZUM KRANKHEITSVERLAUF

Beurteilen Sie den Schweregrad Ihrer COVID-19 Erkrankung anhand folgender Kriterien:

- ○ Ambulant, überwiegend mobil (WHO 1)
- ○ Ambulant, überwiegend bettlägerig (WHO 2)
- ○ Krankenhaus, Normalstation: KEINE Sauerstoff-Nasensonde (WHO 3)
- ○ Krankenhaus, Normalstation: MIT Sauerstoff-Nasensonde (WHO 4)
- ○ Krankenhaus, Intensivstation: KEINE Beatmung (WHO 5)
- ○ Krankenhaus, Intensivstation: MIT Beatmung (WHO 6/7)
- ○ Sonstige, bitte angeben: ______

BEGLEITERKRANKUNGEN

Im Folgenden möchten wir Sie zu Begleiterkrankungen befragen. Gemeint sind Erkrankungen, die Sie bereits vor der COVID-Erkrankung hatten. Zur Übersichtlichkeit sind diese den Organsystemen zugeordnet.

Erkrankungen des Nervensystems

- ☐ Schlaganfall
- ☐ Epilepsie
- ☐ Demenz
- ☐ Polyneuropathie
- ☐ Motoneuronerkrankungen (z.B. Amyotrophe Lateralsklerose)
- ☐ Bewegungsstörung (z.B. Parkinson oder Parkinson-ähnliche Erkrankungen, Kleinhirnerkrankungen)
- ☐ Neuroimmunologische Erkrankungen (z.B.Multiple Sklerose, Myasthenia gravis)
- ☐ Sonstige, bitte angeben: ____________
- ☐ Keine

BEGLEITERKRANKUNGEN

Herz- und Gefäßerkrankungen

- ☐ Bluthochdruck
- ☐ Arteriosklerose
- ☐ Koronare Herzerkrankung
- ☐ Herzinfarkt
- ☐ Rhythmusstörungen
- ☐ Herzinsuffizienz
- ☐ Herzklappenerkrankung
- ☐ Sonstige, bitte angeben: ____________
- ☐ Keine

BEGLEITERKRANKUNGEN

Lungenerkrankungen

- ☐ COPD
- ☐ Asthma
- ☐ Lungenfibrose
- ☐ Sonstige, bitte angeben: ____________
- ☐ Keine

BEGLEITERKRANKUNGEN

Erkrankungen des Magens oder Darms

- ☐ Magengeschwür
- ☐ Entzündliche Darmerkrankung
- ☐ Sonstige, bitte angeben: ____________
- ☐ Keine

BEGLEITERKRANKUNGEN

Erkrankungen der Leber, der Bauchspeicheldrüse oder Diabetes

- ☐ Chronische Lebererkrankung
- ☐ Fettleber
- ☐ Leberzirrhose
- ☐ Diabetes, Tablettentherapie
- ☐ Diabetes, Insulintherapie
- ☐ Entzündung der Bauchspeicheldrüse
- ☐ Unterfunktion der Bauchspeicheldrüse
- ☐ Sonstige, bitte angeben: ______
- ☐ Keine

BEGLEITERKRANKUNGEN

Nierenerkrankungen

- ☐ Chronische Nierenerkrankung
- ☐ Dialysepflichtigkeit
- ☐ Sonstige, bitte angeben: ______
- ☐ Keine

BEGLEITERKRANKUNGEN

Bösartige Erkrankungen

- ☐ Tumorerkrankung
- ☐ Leukämie
- ☐ Lymphom
- ☐ Sonstige, bitte angeben: ____________
- ☐ Keine

BEGLEITERKRANKUNGEN

Rheumatologische Erkrankungen

- ☐ Rheumatoide Arthritis
- ☐ Psoriasis
- ☐ Vaskulitis
- ☐ Lupus erythematodes
- ☐ Dermatomyositis
- ☐ Sonstige, bitte angeben: ____________
- ☐ Keine

BEGLEITERKRANKUNGEN

Allergische Erkrankungen

- ☐ Pollen
- ☐ Hausstaub
- ☐ Medikamente
- ☐ Sonstige, bitte angeben: ________
- ☐ Keine

BEGLEITERKRANKUNGEN

Weitere Vorerkrankungen

- ☐ Organtransplantation
- ☐ Immunsuppression
- ☐ Schilddrüsenüberfunktion
- ☐ Schilddrüsenunterfunktion
- ☐ Hypophyseninsuffizienz
- ☐ Nebenniereninsuffizienz
- ☐ Psychische Erkrankung
- ☐ Suchterkrankung
- ☐ HIV / AIDS
- ☐ Sonstige, bitte angeben: ________
- ☐ Keine

THERAPEUTISCHE MASSNAHMEN

Wurde bei Ihnen wegen der COVID-Erkrankung eine medikamentöse Therapie mit einer der unten aufgeführten Substanzgruppen eingeleitet?

- ☐ Symptomatische Therapie
- ☐ Antibiotikum? (gegen Bakterien)
- ☐ Antimykotikum? (gegen Pilze)
- ☐ Antivirale Therapie? (gegen Viren)
- ☐ Kortison / Steroide
- ☐ Immunsuppressiva
- ☐ Sonstige, bitte angeben: ____________
- ☐ Weiß ich nicht
- ☐ Keine

SARS-COV-2 IMPFUNG

Haben Sie eine COVID-19 Impfung erhalten?

- ○ Ja, EINE Impfdosis am ________ (Format: TT.MM.JJJJ)
- ○ Ja, ZWEITE Impfdosis am ________ (Format: TT.MM.JJJJ)
- ○ Nein

NACHVERFOLGUNG (FOLLOW-UP)

Aufgrund zahlreicher Berichte zu lang anhaltenden Beschwerden möchte wir Sie bitten, Ihre aktuelle Symptomatik anzugeben.
Zur Übersichtlichkeit sind die Symptome erneut nach Organsystemen sortiert. Schauen Sie sich die Liste bitte in Ruhe durch, Mehrfachantworten sind möglich.

Geben Sie hier bitte das heutige Datum ein:

☐ ______ (Format: TT.MM.JJJJ)

Bestehen weiterhin Allgemeinsymptome?

- ☐ Fieber / Schüttelfrost
- ☐ Nachtschweiß
- ☐ Verminderte körperliche Belastbarkeit
- ☐ Sonstige, bitte angeben: ______
- ☐ Keine Symptome

NACHVERFOLGUNG (FOLLOW-UP)

Bestehen weiterhin Beschwerden des Nervensystems?

- ☐ Müdigkeit
- ☐ Schnelle Ermüdbarkeit
- ☐ Konzentrationsschwäche
- ☐ Gedächtnisstörung
- ☐ Schwindel
- ☐ Benommenheit
- ☐ Orientierungsstörung
- ☐ Verwirrtheit
- ☐ Halluzinationen
- ☐ Kopfschmerzen
- ☐ Schlafstörung
- ☐ Alpträume
- ☐ Zittern
- ☐ Krampfanfall
- ☐ Sehstörungen
- ☐ Geruchsstörung
- ☐ Geschmacksstörung
- ☐ Schluckstörungen
- ☐ Sprechstörungen
- ☐ Gangstörungen
- ☐ Lähmungen
- ☐ Taubheitsgefühle
- ☐ Sonstige, bitte angeben: ____________________
- ☐ Keine Symptome

NACHVERFOLGUNG (FOLLOW-UP)

Bestehen weiterhin Beschwerden des Herz und Gefäßsystems?

- [ ] Herzklopfen
- [ ] Herzstolpern
- [ ] Engegefühl in der Brust (Angina pectoris)
- [ ] Herzinfarkt
- [ ] Atemnot in Ruhe
- [ ] Atemnot bei Belastung
- [ ] (Beinvenen-) Thrombose
- [ ] Blutungen
- [ ] Hoher Blutdruck
- [ ] Niedriger Blutdruck
- [ ] Sonstige, bitte angeben: ______
- [ ] Keine Symptome

NACHVERFOLGUNG (FOLLOW-UP)

Haben Sie weiterhin Beschwerden mit den Atemwegen?

- [ ] Laufende Nase (Rhinorrhoe)
- [ ] Verstopfe Nase
- [ ] Halsschmerzen
- [ ] Husten
- [ ] Keuchen
- [ ] Luftnot
- [ ] Lungenentzündung
- [ ] Sonstige, bitte angeben: ______
- [ ] Keine Symptome

NACHVERFOLGUNG (FOLLOW-UP)

Bestehen weiterhin Beschwerden des Magen und Darms oder der Blase?

- ☐ Übelkeit
- ☐ Erbrechen
- ☐ Appetitlosigkeit
- ☐ Bauchschmerzen
- ☐ Durchfall
- ☐ Verstopfung
- ☐ Stuhl-Inkontinenz
- ☐ Harnverhalt
- ☐ Harn-Inkontinenz
- ☐ Sonstige, bitte angeben: ____________
- ☐ Keine Symptome

NACHVERFOLGUNG (FOLLOW-UP)

Haben Sie weiterhin Beschwerden an Haut, Muskel oder Skelettsystem?

- ☐ Hautausschlag
- ☐ Muskelschwäche
- ☐ Muskelschmerzen
- ☐ Gelenkschmerzen
- ☐ Gelenkschwellungen
- ☐ Geschwollene Lymphknoten
- ☐ Geschwollene Beine
- ☐ Sturzneigung
- ☐ Rückenschmerzen
- ☐ Sonstige, bitte angeben: ____________
- ☐ Keine Symptome

NACHVERFOLGUNG (FOLLOW-UP)

Bestehen weitere Symptome?

- ☐ Angststörung
- ☐ Depression
- ☐ Psychose
- ☐ Blutzuckerveränderungen
- ☐ Sonstige, bitte angeben: ______
- ☐ Keine Symptome

BEEINTRÄCHTIGUNG IM ALLTAG

Wie sehr sind Sie aktuell noch durch die COVID-Erkrankung in Ihrem Alltag beeinträchtigt?

Bitte markieren Sie von den untenstehenden 5 Aussagen diejenige, die am stärksten auf Sie zutrifft.

- ○ Ich habe keine Einschränkungen in meinem Alltag und keine Beschwerden, keine Schmerzen, keine Depression und keine Angst mit Bezug auf die Infektion.
- ○ Ich habe vernachlässigbare Beeinträchtigungen in meinem Alltag. Ich kann allen meinen Verpflichtungen und Aktivitäten nachgehen, obwohl ich noch Beschwerden, Schmerzen, Depression oder Angst infolge der Infektion habe.
- ○ Ich bin in meinem Alltagsleben insofern beeinträchtigt, als dass ich durch Beschwerden, Schmerzen, Depression oder Angst infolge der Infektion gelegentlich gezwungen bin, übliche Alltagspflichten oder Aktivitäten zu vermeiden oder zu reduzieren, oder sie über einen längeren Zeitraum zu verteilen. Ich bin aber in der Lage allen meinen Aktivitäten ohne fremde Hilfe nachzugehen.
- ○ Ich leide unter Einschränkungen in meinem täglichen Leben da ich aufgrund von Symptomen, Schmerzen, Depressionen oder Angstzuständen nicht in der Lage bin, alle üblichen Aufgaben/Aktivitäten auszuführen. Ich bin jedoch in der Lage, mich selbst ohne Hilfe zu versorgen.
- ○ Ich leide unter starken Einschränkungen in meinem Alltagsleben: Ich bin aufgrund von Symptomen, Schmerzen, Depressionen oder Angstzuständen nicht in der Lage bin, mich selbst zu versorgen. Ich bin daher auf Pflege und/oder die Hilfe einer anderen Person angewiesen.

KOMMUNIKATIONSBOX

Hier haben Sie die Möglichkeit uns bislang nicht genannte aber für Sie wichtige Punkte mitzuteilen.

Alternativ können Sie direkt Kontakt mit der Forschergruppe per EMail aufnehmen: info@neuro-covid.net

KONTAKTFORMULAR

Möchten Sie über die Studienergebnisse und weitere Aktivitäten der Forschergruppe informiert werden? Beispielsweise zum Einsatz von Wearables (Smartwatches, Fitnesstracker) zur Erkennung von Long COVID?

An dieser Stelle haben Sie die Möglichkeit Ihre Kontaktdaten zu hinterlegen, damit wir Sie auf dem Laufenden halten. Ihre Angaben zu personenbezogenen Daten werden dabei separat und nicht gemeinsam mit den Angaben im Fragebogen gespeichert.

Emailadresse:

Nachname:

Vorname:

Ich möchte an weiteren Umfragen teilnehmen, um die COVID-Forschung zu unterstützen.

- JA
- Nein

Vielen Dank für Ihre Teilnahme!

Wir möchten uns ganz herzlich für Ihre Mithilfe bedanken.

Prof. Dr. Karin Weißenborn, Prof. Dr. Rainer Blasczyk, Dr. Gernot Beutel

Ihre Antworten wurden gespeichert, Sie können das Browser-Fenster nun schließen.
