# Supplementary material for: Brain-binding auto-antibodies in subjects with and without post-COVID-syndrome after SARS-CoV-2 infection
Source: Brain Behav Immun Health. 2026 Sep 18;57:101357. doi: 10.1016/j.bbih.2026.101357 (PMC13625633; doi:10.1016/j.bbih.2026.101357)
Supplement: Supplementary file 2 [file mmc1.docx]

Supplementary Online Content

[Supplementary Table S1. Distribution of presumed SARS-CoV-2 variant periods according to recruitment cohort	2](#_Toc236045581)

[Supplementary Table S2. Multivariable logistic regression analysis of factors associated with NMDAR1 seropositivity	3](#_Toc236045582)

[Supplementary Table S3. Seroprevalence of brain-binding autoantibodies according to recruitment source and PCS status	4](#_Toc236045583)

[Supplementary Table S4. Distribution of PCS patients across PCFS grades	5](#_Toc236045584)

# **Supplementary Table S1. Distribution of presumed SARS-CoV-2 variant periods according to recruitment cohort**

| Cohort | Wild type (2020) | Alpha (Jan–Jun 2021) | Delta (Jul–Dec 2021) | Omicron (2022–2023) |
| --- | --- | --- | --- | --- |
| Plasma donor cohort (n=465) | 93.3% | 6.7% | - | - |
| PCS outpatient cohort (n=148) | 29.8% | 34% | 13.5% | 22.7% |
| Recovered volunteers cohort (n=90) | 20.2% | 2.2% | 2.2% | 75.3% |

Presumed SARS-CoV-2 variant periods were assigned according to the date of infection and the predominant SARS-CoV-2 variants circulating in Germany. Individual viral sequencing data were not available.

# **Supplementary Table S2. Multivariable logistic regression analysis of factors associated with NMDAR1 seropositivity**

| **Variable** | **Adjusted OR** | **95% CI** | **P-value** |
| --- | --- | --- | --- |
| PCS status | 0.754 | 0.49–1.15 | 0.191 |
| Female sex | 0.732 | 0.49–1.10 | 0.135 |
| Age (per year) | 1.033 | **1.01–1.05** | **<0.001** |

Footnote: Odds ratios (ORs) and 95% confidence intervals (CIs) were obtained from a multivariable logistic regression model with NMDAR1 seropositivity as the dependent variable and PCS status, age, and sex as independent variables. Seven participants were excluded because of missing age data (analysis based on n = 696).

# **Supplementary Table S3. Seroprevalence of brain-binding autoantibodies according to recruitment source and PCS status**

|  | **PCS** | |  | **Recovered** | |  |
| --- | --- | --- | --- | --- | --- | --- |
| **Marker** | **Plasma donor**  **PCS n= 281** | **PCS outpatient clinic n= 148** | **P-Value** | **Plasma donor**  **n= 184** | **Recovered volunteers n= 90** | **P-Value** |
| Any AAB, n (%) | 51 (18.1%) | 29 (19.6%) | 0.715 | 31 (16.8%) | 28 (31.1%) | 0.007 |
| NMDAR1, n (%) | 41 (14.6%) | 23 (15.5%) | 0.793 | 26 (14.1%) | 26 (28.9%) | 0.003 |
| NMDAR1-IgA, seropositive, n (%) | 19 (6.8%) | 12 (8.1%) | 0.609 | 10 (5.4%) | 8 (8.9%) | 0.278 |
| NMDAR1-IgM, seropositive, n (%) | 24 (8.5%) | 14 (9.5%) | 0.750 | 17 (9.2%) | 20 (22.2%) | 0.003 |
| NMDAR1-IgG, seropositive, n (%) | 4 (1.4%) | 3 (2.0%) | 0.697* | 2 (1.1%) | 4 (4.4%) | 0.093* |

Values are presented as n (%). Comparisons between recruitment sources were performed separately for participants with PCS and recovered participants using Pearson's χ² tests. P-values marked with an asterisk (*) were calculated using the two-sided Fisher exact test because of small expected cell counts.

# **Supplementary Table S4. Distribution of PCS patients across PCFS grades**

| **PCFS grade** | **Clinical interpretation** | **n (%)** |
| --- | --- | --- |
| 0 | No functional limitations | 146 (34.3%) |
| 1 | Negligible functional limitations | 113 (26.5%) |
| 2 | Slight functional limitations | 102 (23.9%) |
| 3 | Moderate functional limitations | 57 (13.4%) |
| 4 | Severe functional limitations | 8 (1.9%) |
| Total with available PCFS |  | 426 (100.0%) |
| PCFS unavailable |  | 29 |
| Total PCS cohort |  | 455 |

PCFS = Post-COVID Functional Status Score. PCFS data were available for 426 of the 455 PCS patients. Twenty-nine patients from the outpatient cohort could not be included in this analysis because no PCFS assessment was available.
